# Prospective memory instruments for the assessment of children and adolescents: a systematic review

**DOI:** 10.1186/s41155-024-00300-7

**Published:** 2024-05-06

**Authors:** Vanessa de A. Signori, Tiago M. Watanabe, Ana Paula A. de Pereira

**Affiliations:** Human Sciences, Department of Psychology, Federal University of Paraná, Santos Andrade Square 50, Curitiba, Paraná, 80020-300 Brazil

**Keywords:** Prospective memory, Neuropsychological assessment, Neuropsychological instruments, Children, Adolescents, Youth

## Abstract

**Background:**

Prospective memory is the ability to engage in an intention to be performed in the future. The main objective of this study was to identify instruments that assess both time-based and event-based prospective memory in children and adolescents and that have the potential to be clinically applicable.

**Method:**

Three databases (PubMed, Scopus, and PsycINFO) were searched to identify existing PM measures in original articles published until 2022. Literature searches were conducted using the following terms: (prospective memor* OR memor* for intentions) AND (neuropsychological assessment) AND (test* OR instrument* OR questionnaire* OR task*) AND (psychometric properties) AND (child* OR adolescen*). Relevant studies identified in the reference lists were also included in the review.

**Results:**

Ten instruments were identified and classified into three categories: (a) test batteries, (b) experimental procedures, and (c) questionnaires. All the instruments identified were described concerning their content and the psychometric properties available. Some of the instruments presented empirical evidence regarding validity and reliability, but no one provided normative data.

**Conclusion:**

Besides the recent progress regarding studies publishing the development of a variety of novel measures, there are still many limitations surrounding the assessment of PM in the youth population because of the yet incipient psychometric properties presented by the majority of the PM instruments. Recommendations for a gold-standard PM instrument for assessing children and adolescents are provided.

**Supplementary Information:**

The online version contains supplementary material available at 10.1186/s41155-024-00300-7.

Prospective memory (PM) is an umbrella term that designates the abilities engaged in forming an intention to be performed in the future and the processes involved in its execution (Ellis & Freeman, 2008), namely the intention formation, the retention interval, and the retrieval of the intention content (Fish, Wilson, & Manly, 2010). Prospective and retrospective memory (RM) have a close relationship and share some cognitive and psychological processes, such as visual-spatial association, self-processing, and emotion. However, PM has its particularities, which include goal-directed processing, cognitive control, associative learning, and creative thinking (Zheng, Luo, & Yu, 2014). Besides these characteristics, PM tasks are also different from RM tasks because the retrieval of the intention is self-initiated and must happen at a given time or event in the future (Gonneaud et al., 2014), relying on executive processes.

McDaniel and Einstein (2000) have suggested a relevant distinction between two different PM components: event based and time based. The event-based PM corresponds to remembering to perform deferred actions when a particular event occurs, whereas the time-based PM consists of remembering to perform deferred actions at the right moment or within a specified period. This theoretical distinction was further confirmed in brain imaging studies (Gonneaud et al., 2014; Momennejad & Haynes, 2012; Okuda et al., 2007). Gonneaud et al. (2014) assessed PM using functional magnetic resonance imaging and reported that the constant target checking related to event-based conditions is supported by stronger occipital activation, whereas time-based conditions have periodic monitoring revealed by a right-sided frontal network. Besides the proposition of these two components, McDaniel and Einstein (2000) have also suggested a variety of critical factors that are expected to affect the strategies used by individuals to remember the actions that need to be performed in the future, which include the importance of the PM task itself, characteristics of the retrieval cues and their relation to the target actions, the nature of the ongoing task, socio-environmental context, and individual characteristics.

Assessing PM performance in children and adolescents may enable clinical neuropsychologists to better identify neurodevelopmental disorders and prescribe more accurate rehabilitation procedures. However, instruments developed specifically to assess PM in the youth population are still scarce, and, consequently, this cognitive domain is not regularly assessed in clinical neuropsychological testing. With that in mind, the current systematic review aimed to (a) identify the available measures to assess both time-based and event-based PM abilities in children and adolescents and (b) describe their content and psychometric properties (when available). Our main goal is to assist clinical neuropsychologists by providing them with knowledge on the instruments available for assessing PM in children and adolescents and the validity and reliability properties presented by each one of them.

## Method

A protocol for this study was recorded on the International Prospective Register of Systematic Reviews (PROSPERO) by the register CRD42022334051. The execution and the reporting of this review are consistent with the Preferred Reporting Items of Systematic Reviews and Meta-Analyses (PRISMA) guidelines (Moher et al., 2009; Rethlefsen et al., 2021).

To our knowledge, this is the first study to objectively assemble and screen the literature in search of PM instruments for the clinical assessment of children and adolescents. To accomplish this objective, we selected empirical studies that met the following criteria: (a) were published in English or Portuguese, (b) were published in peer-reviewed journals, (c) were experimental studies that assessed both event-based and time-based PM abilities using one single instrument, and (d) the assessment was conducted in children or adolescents up to the age of 19. Studies were excluded if they (a) were primarily focused on a cognitive domain other than PM, (b) did not consider PM as an isolated construct, (c) were studies focusing on PM training or rehabilitation, and (d) were single case studies, review articles, systematic reviews, or meta-analyses. All studies that met the criteria were included in the review.

A systematic search of published articles was conducted in June 2022 on PubMed, Scopus, and PsycINFO databases. The following terms were searched: (prospective memor* OR memor* for intentions) AND (neuropsychological assessment) AND (test* OR instrument* OR questionnaire* OR task*) AND (psychometric properties) AND (child* OR adolescen*). Searching in the PubMed database followed a different method: all of the terms were used without the symbol for truncation (*), since, specifically in this database, this method resulted in a higher number of findings. All of the articles found were screened based on their titles and abstracts. The articles found in the databases aforementioned were screened independently by two researchers using the Abstrackr machine learning tool (Wallace, Small, Brodley, Lau, & Trikalinos, 2012). The double-screen mode was chosen, which means that each abstract was screened once by each one of the reviewers. Meanwhile, the studies found either in the article’s reference lists or in other sources were screened independently by the same two researchers using an Excel spreadsheet.

The identified PM measures were assigned to three distinct types of assessment: test batteries, questionnaires, and experimental procedures. The classification followed the rationale proposed by Blondelle, Hainselin, Gounden, and Quaglino (2020) previous review of PM instruments. Data extraction of each study was also conducted independently by the same two researchers and included the name of the tests, the name of the first author, publication year, sample’s characteristics (age range with mean and standard deviation, number of participants, and their description), type of measures, authors’ hypotheses, study’s description, the other neuropsychological measures administered in the assessment session(s), and the psychometric properties available in the paper. All of the PM instruments that were considered to be test batteries were also described considering their estimated duration, retention intervals, number of PM items, response modalities, ongoing task, scoring information, recognition task, and qualitative measures (if available).

Evaluation of the methodological quality was carried out using the COSMIN Risk-of-Bias checklist (Mokkink et al., 2018), which assesses each instrument under 10 domains: patient-reported outcome measures development, content validity, structural validity, internal consistency, cross-cultural validity/measurement invariance, reliability, measurement error, criterion validity, hypotheses testing for construct validity, and responsiveness. Considering that all of the instruments reviewed in the present study were designed with the same comprehension of the PM construct in mind, it was decided to exclude the first two components of the COSMIN checklist. In the COSMIN user manual, it is suggested for the review team not to use the boxes for criterion validity and responsiveness in the systematic review when no gold standard measurement is available (Mokkink et al., 2018, p.40); therefore, it was decided to exclude these items from the present review. The measurement error and the hypotheses testing domains were also excluded from this review since no study reported this data and that no hypotheses were defined by the review team for each study.

## Results

**Fig. 1:**
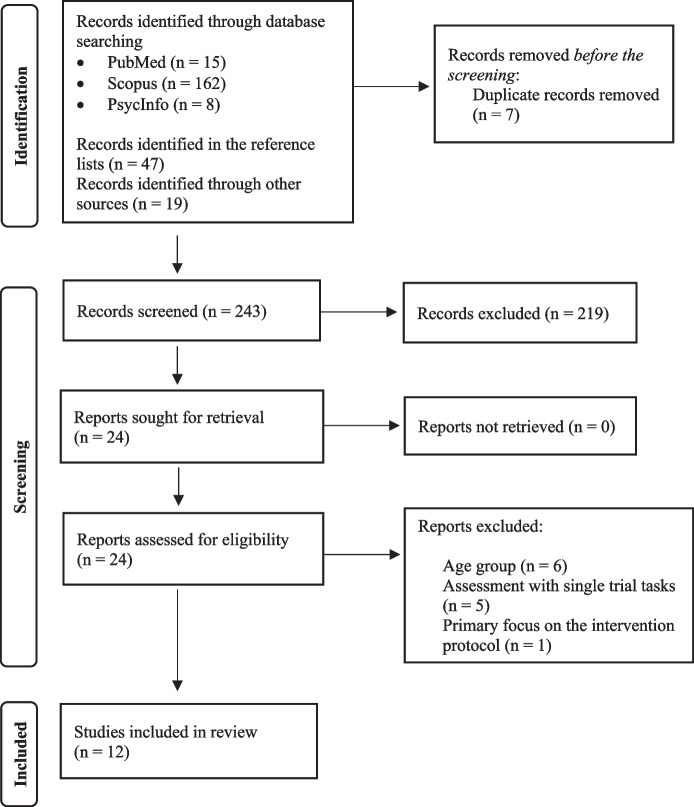
Flow chart depicting the study selection process through the phases of the systematic review

The initial literature search generated 184 articles. Sixty-six studies were further identified in the reference lists or other Internet sources and were also screened based on their titles and abstracts. From the 250 preliminary reports, 7 duplicates were removed, and 219 were excluded. Cohen’s kappa (Cohen, 1960) for two raters was conducted to verify the reliability of the screening process and presented results of good agreement between the two researchers (*k* = 0.726, *p* < .001). The resolution of the conflicts was conducted by the same two researchers after discussing each abstract until an agreement was reached. After reviewing the entire content of 24 articles and applying the exclusion criteria, the number of studies that met the inclusion criteria and were included in the review was 12 (see Fig. 1 for an overview of the selection process).

In this review, 10 PM measures were identified and assigned to three distinct types of assessment: (a) five test batteries: the PROMS (Osipoff, Dixon, Wilson, & Preston, 2012), the prospective memory trials (Robey, Buckingham-Howes, Salmeron, Black, & Riggins, 2014), the Prospective Memory Assessment for Children & Youth (Garvie et al., 2019), the Memory for Intention Screening Test for Youth (Mills, Raskin, & DiMario, 2020), and the PM Test (Stedall et al., 2022); (b) three experimental procedures: the Happy Week (Yang, Chan, & Shum, 2011), the Virtual Week (Henry et al., 2014), and the Paperboard PM Tasks (Han et al., 2017); and (c) two questionnaires: the Prospective and Retrospective Memory Questionnaire for Children (Talbot & Kerns, 2014) and the Children’s Future Thinking Questionnaire (Mazachowsky & Mahy, 2020). Information regarding each one of the studies included in this review is available in Table 1, and each instrument’s psychometric properties are available in the supplementary material.

**Table 1:** Summary of studies characteristics

Authors, year	Instrument	*N*	Age	Sample	Other neuropsychological tests	Conclusions
Yang et al., 2011	Happy Week	120	7–12	Non-clinical	The short version of the WISC-Chinese, n-back task; Walk Don’t Walk from TEA-Ch, Fishing Game	Age was considered a significant factor in increasing PM scores. The correlation between the two PM tasks was considered modest but significant for both total scores and subscales. IQ score was found to correlate to most PM scores even after controlling for age
Osipoff et al., 2012	PROMS	94	12.5 (3.4)	Type 1 diabetes mellitus	WASI (2-subtest), WIAT-2, CVLT-C, digit subtest from WISC-IV, and BRIEF	PROMS scores did not correlate with children’s performance on the other cognitive tests. No association between total PROMS score and parental ratings on the BRIEF was found. The only significant correlation between HbA1C and PROMS was the 20-min event-based task
Robey et al., 2014	PM Trials	105	15.45 (1.07)	PDE and CG	Color-Word Interference Test D-KEFS, CPT-II, Spatial Working Memory task from CANTAB, CVLT-C, WASI (2-subtest), and BRIEF	No differences were found between PDE and CG on PM scores and other cognitive measures. Significant correlation between PM scores and adolescents’ performance in RM and EF tests were found
Henry et al., 2014	Virtual Week	60	10.05 (1.46)	ASD and CG	SCQ, WASI, Trail Making Test, Verbal Fluency, and the Color Word Inference subtests from the D-KEFS, ABAS-II	For the ASD group, better performance on the time-based score was correlated with IQ, EF, and functional independence. For the CG group, both event- and time-based scores were correlated with EF
Talbot & Kerns, 2014	PRMQC	69	10.87 (1.64)	ADHD and CG	WASI, Super Little Fisherman; CyberCruiser-II, Conner’s ADHD Index	Age was a significant predictor of time-based PM. Control group significantly outperformed ADHD group for event-based and time-based PM tasks. PRMQC was significantly correlated with intellectual ability (WASI full scale) and with Conner’s ADHD Index
Han et al., 2017	Paperboard PM task	I. 105 II. 103 III. 106	3–5	Non-clinical	-	Five-year-old children were significantly better than the younger children. High level of OT’s difficulty resulted in worse PM scores, even in a high-motivation condition
Harris et al., 2017	PROMACY	258	14.2	PHEU, PHIV/C, and PHIV/non-NCI	NEPT, WRAML2, WISC-IV or WAIS-IV, D-KEFS	PHIV/C scores were significantly lower than PHIV/non-C and PHEU on both PROMACY and NEPT scores. NEPT has presented increased sensitivity when compared to PROMACY
Garvie et al., 2019	PROMACY	54	13 (2.6)	PHEU	NEPT, WRAML-2, WISC-IV or WAIS-IV, and D-KEFS	PROMACY presents acceptable internal consistency and split-half reliability. Correlation with NEPT was not significant
Mazachowsky & Mahy, 2020	CFTQ	I. 145 II. 255 III. 101 IV. 105	3–7	Non-clinical	Picture-Book task, truck loading task, choice delay, PM event-based task, marble game, Simon Says, PPVT-IV, future-preferences task, Saving Board Game, Tower of Hanoi, Vehicle Card Sord task, Gift Delay, Dimensional Change Card Sort Test and Picture Vocabulary Test from the NIH-TB, BRIEF-P	Across all studies, the CFTQ has demonstrated high internal consistency on all of the five subscales as well as the full scale. However, the PM subscale was not significantly correlated with the PM task and with parental report on their children’s PM ability
Mills et al., 2020	MISTY	124	9.57 (2.86)	Non-clinical	-	MISTY presents acceptable internal consistency and split-half reliability
Mills et al., 2021	MISTY	45	9.6 (1.5)	IE and CG	KBIT-2 and the PM items from the RBMT-C	No differences were found between IE and CG on PM scores measured by MISTY. Significant differences between groups were found in RBMT-C total score
Stedall et al., 2022	PM Test	107	13.3 (0.35)	VP and CG	Dot locations and word pairs subtests from CMS, WASI (2-subtest), and OMQ-P	VP total scores were significantly lower than CG. The VP group performed poorer than the CG on time-based and short-term tasks

*WISC* Wechsler Intelligence Scale for Children; *TEA-Ch* Test of Everyday Attention for Children-Chinese version; *PM* prospective memory; *IQ* intelligence quotient; *WASI* Wechsler Abbreviated Scale of Intelligence; *WIAT-2* Wechsler Individual Achievement Test-Second Edition; *CVLT-C* California Verbal Learning Test-Children’s Version; *BRIEF* Behavior Inventory of Executive Functions; *HbA1C* hemoglobin A1C; *PDE* prenatal drug exposure; *CG* control group; *D-KEFS* from Delis-Kaplan Executive Functioning System; *CPT-II* continuous performance test; *CANTAB* Cambridge Neuropsychological Test Automated Battery; *RM* retrospective memory; *EF* executive functions; *ASD* autism spectrum disorder; *SCQ* Social Communication Questionnaire; *ABAS* Adaptative Behavior Assessment Scale; *PRMQC* Prospective Retrospective Memory Questionnaire for Children; *ADHD* attention-deficit hyperactivity disorder; *OT* ongoing task; *PROMACY* Prospective Memory Assessment for Children & Youth; *PHEU* perinatally HIV-exposed uninfected; *PHIV/C* perinatally HIV infected with neurocognitive impairment; *PHIV/Non-C* perinatally HIV infected without neurocognitive impairment; *NEPT* naturalistic event-based prospective memory task; *WRAML-2* Wide Range Assessment of Memory Learning-2nd Edition; *WAIS* Wechsler Adult Intelligence Scale; *CFTQ* Children’s Future Thinking Questionnaire; *PPVT-IV* Peabody Picture Vocabulary Test; *NIH-TB* National institutes of Health Toolbox for the Assessment of Neurological and Behavioral Function; *MISTY* Memory for Intentions Screening Test for Youth; *IE* idiopathic epilepsy; *KBIT-2* Kaufman Brief Intelligence Test-2nd Edition; *RBMT-C* Rivermead Behavioral Memory Test for Children; *VP* very preterm; *CMS* Children’s Memory Scales; *OMQ-P* Observer Memory Questionnaire-Parent Form

### Test batteries

PROMS (Osipoff et al., 2012) is one of the instruments that were adapted especially for the pediatric population. Due to the absence of a gold standard instrument to assess PM in children and adolescents, the authors developed a modified version of a previously published adult screening test. Children and adolescents with type 1 *diabetes mellitus* were asked to complete the test to investigate the relationship between PM performance and poor glycemic control, which was measured by higher hemoglobin A1C (HbA1c) value. Other four cognitive tests were used to estimate general intelligence, academic abilities, declarative memory, and working memory. In addition to the performance tests, the Behavior Inventory of Executive Functions — BRIEF (Gioia et al., 2000; apud Osipoff et al., 2012) was also completed by the participant’s parents. According to the authors, regression analysis has indicated that lower scores on the 20-min event-based task (EBT) were able to predict higher HbA1c values (*β* = −0.22, *p* < .05) (Table 2).

**Table 2:** Characteristics of the five PM Test batteries included in the review

Instrument	PROMS	PM Trials	PROMACY	MISTY	PM Test
First author	Osipoff, J. N.	Robey, A.	Garvie, P. A.	Mills, G. N.	Stedall, P. M.
Year	2012	2014	2019	2021	2022
Time duration	50 min	NI	20 min	20 min	NI
Practice trial	No	Yes	No	No	Yes
TB tasks	4	2	4	4	4
EB tasks	4	3	4	4	4
Response modalities	V/A	A	V/A	V/A	V/A
Ongoing task	Academic tests	Questionnaire and CT	Word-search puzzle	Word-search puzzle	CT
Total score	0–16	0–10	0–48	0–16	0–16
Recognition task	No	No	Yes	Yes	No
Delayed intervals (min)	2, 5, 10, 15, and 20	2 and 15	2 and 10	2 and 10	ST: 5, 10, 15, 45 and LT*
Qualitative measures	NI	NI	OM, TS, LOC, LOT, PLO, RD	PF, TS, LOC, LOT, RD	NI

*NI* not informed; *TB* time based; *EB* event based; *V/A* verbal and action; *A* action; *CT* cognitive tests; *ST* short term; *LT* long term; *OM* omission; *TS* task substitution; *LOC* loss of content; *LOT* loss of time; *PLO* place losing omission; *RD* random; *PF* prospective failure. *Long-term tasks must be held on the same day at night and 1 week after

The Prospective Memory Trials (Robey et al., 2014) was  based on the Memory for Intentions Screening Test — MIST (Raskin, 2004; Raskin, 2009). The instrument was used to evaluate 59 prenatally drug-exposed (PDE) adolescents and 46 healthy controls (HC). The study aimed to examine how PM relates to other cognitive abilities and to the subject’s brain structure, which was measured by magnetic resonance imaging. Analyses of covariance (ANCOVAs) revealed no differences between PDE and HC neither in PM performance nor on the other cognitive measures. Despite these results, adolescents with a history of PDE evidenced poorer executive function reported by their caregivers on the BRIEF (Gioia et al., 2000; apud Robey et al., 2014). PDE and control group results were combined and indicated a significant correlation between PM scores and adolescents’ performance in RM and EF tests.

The Prospective Memory Assessment for Children & Youth (PROMACY) was also developed based on the MIST and was designed for use with children and adolescents from 8 to 21 years old (Garvie et al., 2019). The instrument was pilot-tested with a small sample of 29 subjects with a mean age of 12.1 years old (*SD* = 2.7; range 8–17) to obtain preliminary psychometric properties. After demonstrating promising results, the validation study was conducted with a sample of 54 perinatally HIV-exposed uninfected (PHEU) subjects. As reported by the authors, PROMACY’s internal consistency was low but acceptable (*α* = 0.60), mainly considering it is a short-item instrument. The Spearman-Brown coefficient for split-half reliability was 0.67, and Cronbach’s *α* coefficient for subscale scores ranged between 0.22 and 0.64. Despite the absent correlation between PROMACY’s scores and the naturalistic event-based prospective memory task (NEPT), children’s performance in PROMACY’s scores was associated with their own performance on IQ, RM, WM, and EF tests. A previous memory study (Harris et al., 2017) also combined data from PROMACY and NEPT as PM measures to evaluate 85 perinatally HIV-exposed uninfected (PHEU), 45 perinatally HIV infected with neurocognitive impairment (PHIV/NCI), and 128 perinatally HIV infected without neurocognitive impairment (PHIV/non-NCI). Even though NEPT has presented increased sensitivity when compared to PROMACY’s results, both instruments were sensitive to PHIV/NCI cognitive deficits.

More recently, the MIST (Raskin, 2004; Raskin, 2009) was adapted for use in children and adolescents and published as the Memory for Intentions Screening Test for Youth — MISTY (Mills et al., 2020). The psychometric study of the MISTY was conducted with a nonclinical sample of 124 children and adolescents from 4 to 15 years old. According to the authors, inter-item reliability was considered good, and the six subscales’ scores revealed a high level of internal consistency (*α* = 0.87). Split-half reliability was measured by the Spearman-Brown coefficient, and the intraclass correlation coefficient was used to investigate the reliability between the two raters who independently scored the test forms. Comparison between subgroups was further conducted in children with idiopathic epilepsy and healthy controls, and the authors reported that there were no significant differences in the MISTY total score, subscale scores, recognition task, or ongoing task (Mills, Garbarino, & Raskin, 2021).

The prospective memory test (Stedall et al., 2022) is an instrument recently published and developed based on the MIST, the Royal Prince Alfred Prospective Memory Test — RPA-ProMem (Radford, Lah, Say, & Miller, 2011), and the Rivermead Behavioral Memory Test — RBMT (Wilson et al., 2008). The instrument was tested in 81 adolescents born very preterm (< 30 weeks’ gestational age) and 26 healthy controls. The study aimed to investigate episodic and prospective memory in this clinical population. In terms of PM measures, the clinical group performed poorer than the control group on total scores, time-based, and short-term scores; thus, PM performance evidenced a moderate-to-strong effect in between-group analysis.

### Experimental procedures

Two experimental procedures were developed in the same study to compare event-based, time-based, and activity-based PM measures in children. The Fishing Game was described by the authors (Yang et al., 2011) as a computer game in which children are required to hook as many fish as possible meanwhile feeding the cat next to the boy sitting on a boat whenever a cue is detected. In this experimental procedure, event-based and time-based cues were counterbalanced as two separate conditions; therefore, this instrument was not considered in this review. On the other hand, Happy Week is a board game designed to simulate real-life tasks that are usually done within a week. In this instrument, three tasks need to be completed in regular intervals and three tasks to be completed occasionally while moving a toy car from the start to the end of a virtual day. A nonclinical sample of 120 children from 7 to 12 years old was assessed with both instruments together and with 3 other cognitive tests. The two experimental procedures developed for this study demanded different response modalities (motor response in the Fishing Game and verbal response in the Happy Week) and different levels of difficulty in recalling the intended actions. These factors are argued by the authors to be the reasons why the correlation between these two instruments was considered low after controlling for age. Nonetheless, both experimental procedures were found to be sensitive instruments to capture PM development in children.

Virtual Week (Henry et al., 2014) was adapted from its original version which was developed for use with adults (Rendell & Craik, 2000). In this modified version, the authors adapted the ongoing activities, and the PM tasks themselves to be more pertinent to children’s everyday life. To advance the literature about PM performance in neurodevelopmental disorders, 30 children with ASD and a comparison group of 30 typically developing children were evaluated. All of the 60 children ranging from 8 to 12 years old were assessed in their own homes with two versions of the Virtual Week. In addition, participants were also evaluated with global intelligence and executive function tests. Parents were given the Adaptative Behavior Assessment Scale — ABAS-II (Harrison & Oakland, 2003; apud Henry et al., 2014), and the Social Communication Questionnaire — SCQ (Rutter et al., 2003; apud Henry et al., 2014), to complete during their children’s test session. The reliability of the Monday to Wednesday version of the Virtual Week was *α* = 0.84 for the control group and *α* = 0.58 for ASD, whereas the Thursday to Saturday version was *α* = 0.78 for the control group and *α* = 0.57 for ASD. According to the authors, these results demonstrate a strong reliability in the use of Virtual Week for assessing typically developing children and a moderate reliability for the assessment of children with ASD.

The Paperboard PM Task (Han et al., 2017) was another experimental procedure developed to investigate three different objectives: (1) the developmental trajectory of preschool children in both time-based and event-based PM tasks, (2) the influence of the ongoing task difficulty on children’s PM performance, and (3) the influence of the ongoing task difficulty on children’s PM performance in the context of increased motivation. In the first experiment, children were presented with the paperboard ongoing task and instructed about the PM tasks inserted on it. After practicing the execution, children were presented with a 3-min interference task. The second experiment was conducted identically to the first, except for the additional complexity of the ongoing task. The third experiment’s methodological procedure was identical to the one employed in the second experiment. However, one additional instruction was given to the children in order to increase their motivation for seeking a better performance on the ongoing task. A series of between-group analyses have demonstrated that PM performance measured by the Paperboard PM Task is sensitive to levels of difficulty on the ongoing task and age differences.

### Questionnaires

The Prospective Retrospective Memory Questionnaire for Children — PRMQC (Talbot & Kerns, 2014) is a 16-item brief report of memory failures in everyday life adapted to be completed by children’s and adolescents’ parents based on a self-report original version developed for young and older adults by Smith, Del Sala, Logie, and Maylor (2000). The authors aimed to investigate PM performance in children with ADHD using a time reaction (TR) task, the PRMQC parent report, and two separate PM tasks: the Super Little Fisherman event-based PM task (Yang et al., 2011) and the CyberCruiser-II time-based PM task (Kerns, 2000). The study was conducted with a total sample of 69 children with and without ADHD ranging from 8 to 13 years old. All participants completed the aforementioned tasks, while parents were given the PRMQC to complete during their children’s testing session. According to Talbot and Kerns (2014), the control group (*n* = 33) significantly outperformed the ADHD group (*n* = 36) in the event-based and time-based PM tasks. For PRMQC, Cronbach’s *α* coefficient for total score was 0.93, for prospective scale was 0.91, and for retrospective scale was 0.81. Parents’ reports in the PRMQC were also significantly correlated with their children’s performance in IQ, time-based, and event-based PM tasks.

The Children’s Future Thinking Questionnaire — CFTQ (Mazachowsky & Mahy, 2020) is a 44-item parent report that assesses their children’s abilities of saving, planning, delaying gratifications, episodic foresight, and PM. This instrument was initially developed with 79 items, and, in the first study, the authors provided evidence for its reliability. The second and third studies involved the refinement of the scale to a shorter version containing 44 items and its investigation in terms of validity and reliability. The fourth study evidenced excellent test-retest reliability. Across all studies, the CFTQ demonstrated high internal consistency on all five subscales and the full scale. Regarding validity, the CFTQ PM subscale was not correlated with the PM performance-based tasks, although it was significantly correlated with parent reports on the BRIEF-P memory items.

## Discussion

According to our knowledge, this is the first review aiming to identify the available measures that assess both time-based and event-based PM abilities and that are specifically designed for use in children and adolescents. In this review, 10 instruments were identified and categorized as test batteries, experimental procedures, or questionnaires. All of the instruments found in the selected databases were described concerning their content and were rated based on the COSMIN Risk-of-Bias checklist (Mokkink et al., 2018). As described in Table 3, the instrument’s rating criteria suggest a very low quality of evidence regarding their use in the PM assessment of children and adolescents.

**Table 3:** Rating criteria of the test instruments included in the review

Instrument	Structural validity	Internal consistency	Reliability	Cross-cultural validity
Happy Week	-	?	-	-
PROMS	-	?	-	-
PM Trials	-	?	-	-
Virtual Week	-	?	-	-
PRMQC	-	?	-	-
Paperboard PM Task	-	?	-	-
PROMACY	-	?	-	-
CFTQ	+	+	-	-
MISTY	-	?	+	-
PM Test	-	?	-	-

The measurement property of the instrument is considered sufficient (+), insufficient (-), inconsistent (±), or indeterminate (?)

Concerning the PROMS study (Osipoff et al., 2012), it is important to highlight that the absent data from healthy controls restricted the validity analysis, and the correlation analyses with other instruments failed to demonstrate any relation between PM scores and the subject’s performance on the standardized cognitive tests or parental ratings on the BRIEF. Another characteristic that needs to be pointed out as a possible limitation of its application in clinical settings is the extensive time to execute the task (50 min). Regardless of the issues abovementioned, PROMS still seems to be a promising instrument to assess PM in children and adolescents because of its adequate theoretical basis.

Contrarily to PROMS, in the PM Trials study (Robey et al., 2014), the authors reported a between-group analysis with a clinical sample being compared to healthy controls. Despite failing to demonstrate differences between PDE and the control group in PM performance, significant correlations between PM scores and adolescents’ performance in RM and EF tests were found. Aside from its convergent validity, PM Trials is the only instrument that requires uniquely motor actions as responses to PM tasks and, due to that characteristic, should be considered by clinicians as the first option for assessing PM in children and adolescents with speech or other language impairments. However, the imbalance in the number of time-based tasks (TBT) in relation to EBT must be mentioned as a weak point and could affect the vastness of its use in the clinical setting.

As far as we know, PM Test is the latest paper-and-pencil instrument that was developed specifically for the assessment of PM in children and adolescents and was published in the international scientific literature. Stedall et al. (2022) reported that PM Test was sensitive in revealing PM deficits in a between-group analysis comparing very preterm children (below 30-week gestational age) and the control group. As a unique characteristic, the prospective memory test is the only instrument that contains a long-term task (1-week interval after the session) to be performed outside of the clinical setting. This attribute emulates adult instruments for assessing PM and can offer a measure for a naturalistic context.

In opposition to the previous instruments, PROMACY demonstrated internal consistency and split-half reliability properties, yet, in the event-based scale, limited reliability and notable ceiling effects were presented. According to Garvie et al. (2019), a possible contributor to these problems may be the fourth-grade level of difficulty which was selected to be sufficiently broad for all age groups evaluated but ended up being an exceptionally easy stimulus for the older subgroup of participants. In the between-group analysis, NEPT presented increased sensitivity when compared to PROMACY’s results. Nonetheless, both instruments were sensitive to PHIV/NCI cognitive deficits. Besides these preliminary validity and reliability results, PROMACY offers qualitative measures that can add valuable information to clinicians.

Similarly to PROMACY, MISTY (Mills et al., 2021) presented data for internal consistency and split-half reliability. A comparison between a clinical group of children with idiopathic epilepsy and healthy controls (Mills et al., 2021) was also conducted even though differences in their performances were not found. Despite the absent sensitivity in acknowledging PM deficits in this sample, MISTY has presented promising psychometrics results. It also should be highlighted that MISTY is the only instrument in which time-based tasks anticipate information about the exact time at which intentions are expected to be executed and, therefore, can diminish possible failures related to deficits in arithmetic abilities. In addition to this important characteristic, MISTY also offers qualitative measures and, consequently, appears to be one of the best options for evaluating PM in clinical assessments.

In summary, of the five test batteries included in this review, only the prospective memory trials (Robey et al., 2014) contain a different number of time-based and event-based tasks and proposed only one type of response modality (action). In relation to the delayed intervals, only the prospective memory test (Stedall et al., 2022) proposed a long-term PM task. Otherwise, both prospective memory trials and prospective memory test contain a practiced trial to be held before the formal PM evaluation. Meanwhile, the PROMACY (Garvie et al., 2019) and the MISTY (Mills et al., 2020) are the only instruments that incorporated recognition tasks and qualitative measures to investigate the types of errors carried out during the assessment. However, all five paper-and-pencil instruments require writing skills to accomplish intended actions, which is an obstacle for assessing PM in children and adolescents with learning disabilities related to written language.

## Conclusions

There has been an increasing interest in the human development of both time-based and event-based PM (Talbot & Kerns, 2014), yet the majority of articles published to date have relied on their results on single-trial tasks (Mills et al., 2021). Overall, it is possible to evidence that, besides the recent progress derived from the development of a variety of novel PM measures, there are still many limitations surrounding the assessment of PM in the youth population because of the incipient psychometric properties presented by the majority of PM instruments. Research on instruments for assessing PM in children and adolescents is important for increasing the knowledge surrounding PM human development and providing psychometrically sound instruments for future clinical assessment of PM in youth.

The previously mentioned findings raise some interesting theoretical questions: (1) Besides increasing age, are there other sociodemographic variables that play an important role in PM development? and (2) if age is the main predictor for PM development during childhood and adolescence, then should all instruments be designed considering different levels of complexity and for their use in a small age group rather than a broad range of age groups? As has already been stated by Mills et al. (2021), future studies investigating age groups and comparing their PM performance to the development of their brain and other cognitive processes could provide information for a better understanding of PM development and, consequently, its assessment. Until then, findings from recent studies suggest the need for specific age-based norms.

This review intended to provide clinical neuropsychologists with knowledge surrounding the instruments developed for the assessment of PM in children and adolescents and summarize their validity and reliability properties. Some of the specified features highlighted in each one of the instruments could be considered recommendations for a gold-standard PM instrument, particularly the presence of a practice trial before formal PM assessment, the counterbalanced number of time-based and event-based tasks, and the inclusion of a recognition task and a qualitative measure such as error type. In order to provide a broader range of children and adolescents with the conditions to be properly evaluated, we also suggest that further studies on the development of PM instruments avoid written skills as a prerequisite for fulfilling PM intentions.

### Limitations of this review

We acknowledge that the current review has some limitations. Firstly, we did not include any instrument considered a single-task PM measure. Furthermore, the search terms used to screen for studies in the databases can be considered overly restrictive, and, in consequence, a small number of studies have been selected for inclusion in this review. Indeed, we may have forsaken some experimental and naturalistic instruments. This decision, though, relates to our main goal to provide clinicians with the best possible options to evaluate PM as an isolated and undivided construct in a clinical setting.

### Supplementary Information

**Additional file 1:.** Supplementary tables: Supplementary Table 1: Psychometric properties of the test batteries included in the review. Supplementary Table 2: Psychometric properties of the experimental procedures included in the review. Supplementary Table 3: Psychometric properties of the questionnaires included in the review.

## Data Availability

Not applicable

## Abbreviations

ADHD: Attention-deficit/hyperactivity disorder
ASD: Autism spectrum disorder
BRIEF: Behavior Inventory of Executive Functions
CFTQ: Children’s Future Thinking Questionnaire
EBT: Event-based task
EF: Executive functions
HC: Healthy controls
MIST: Memory for Intentions Screening Test
MISTY: Memory for Intentions Screening Test for Youth
NEPT: Naturalistic event-based prospective memory task
PDE: Prenatally drug-exposed
PHEU: Perinatally HIV-exposed uninfected
PHIV-NCI: Perinatally HIV-infected with neurocognitive impairment
PHIV/non-NCI: Perinatally HIV-infected without neurocognitive impairment
PM: Prospective memory
PRMQC: Prospective Retrospective Memory Questionnaire for Children
PROMACY: Prospective Memory Assessment for Children & Youth
RM: Retrospective memory
TBT: Time-bases task
WM: Working memory
