# Supplementary material for: Prospective memory instruments for the assessment of children and adolescents: a systematic review
Source: Psicol Reflex Crit. 2024 May 6;37:17. doi: 10.1186/s41155-024-00300-7 (PMC11074089; doi:10.1186/s41155-024-00300-7)
Supplement: Supplementary file 1 — Additional file 1:. Supplementary tables: Supplementary Table 1: Psychometric properties of the test batteries included in the review. Supplementary Table 2: Psychometric properties of the experimental procedures included in the review. Supplementary Table 3: Psychometric properties of the questionnaires included in the review. [file 41155_2024_300_MOESM1_ESM.docx]

**Supplementary Table 1**

*Psychometric properties of the test batteries included in the review*

| PSYCHOMETRIC PROPERTIES | PROMS (2012) | PM TRIALS (2014) | PROMACY (2019) | MISTY (2020) | PM TEST (2022) |
| --- | --- | --- | --- | --- | --- |
| Structural Validity | - | - | - | - | - |
| Internal Consistency | - | - | α = .60 | α = .87 | α = .77 |
| Split-half Reliability | - | - | r = .67 | r = .89 | - |
| Inter-rater Reliability | - | - | - | ICC = 1.00 | - |
| Test-Retest | - | - | - | - | - |
| Criterion Validity | - | - | NEPT (p > .10) | - | - |
| Convergent Validity | - | CVLT-C (r =.24-.43) D-KEFS (r =.28-.32) BRIEF (r =.22) | WISC/WAIS (r =.45) D-KEFS (r =.30-.46) WRAML-2 (r =.38-.39) | - | - |
| Predictive Validity | EBT predicted HbA1c (β = -.22, p < .05) | - | - | - | - |
| Between-group Analysis | - | - | PHIV/C < PHIV/Non-C, and PHEU (p <.001) | CG – IEG  (p = .276) | VP < CG (p =.01) |

*Note.* EBT = Event-based task; HbA1c = hemoglobin A1C; CVLT-C = California Verbal Learning Test - Children's Version; D-KEFS = Delis-Kaplan Executive Functioning System; BRIEF = Behavior Inventory of Executive Functions; NEPT = Naturalistic Event-Based Prospective Memory Task; WISC = Wechsler Intelligence Scale for Children; WAIS = Wechsler Adult Intelligence Scale; WRAML-2 = Wide Range Assessment of Memory Learning – 2^nd^ edition; VP = very preterm; PHIV/C = perinatally HIV infected with neurocognitive impairment; PHIV/non-NCI = PHIV perinatally HIV infected without neurocognitive impairment; PHEU = perinatally HIV-exposed uninfected; ICC = Intraclass Correlation; CG = Control Group; IEG = Idiopathic Epilepsy Group; VP = Very Preterm.

**Supplementary Table 2**

*Psychometric properties of the experimental procedures included in the review*

| PSYCHOMETRIC PROPERTIES | HAPPY WEEK (2011) | VIRTUAL WEEK (2014) | PAPERBOARD PM TASK (2017) |
| --- | --- | --- | --- |
| Structural Validity | - | - | - |
| Internal Consistency | - | ASD group: MWV α = .58; TSV α = .57  Control group: MWV α = .84; TSV α = .78 | - |
| Split-half Reliability | - | - | - |
| Inter-rater Reliability | - | - | - |
| Test-Retest | - | - | - |
| Criterion Validity | Fishing Game (r =.38) | - | - |
| Convergent Validity | N-Back Task (r =.36) Walk don’t Walk (r =.36) WISC (r =.43) | ASD group: WASI and TB score (r =.51); CWI and TB score (r =.47); SCQ and TB score = (r =.57)  Control Group: VF score (r =.43-.54); SCQ and TB score (r =.45) | - |
| Predictive Validity | - | - | - |
| Between-group Analysis | - | TB score: ASD < CG (p <.001) | 5-year-old > 4-year-old (p < .05)  4-year-old > 3-year-old (p < .001) |

*Note.* WISC = Wechsler Intelligence Scale for Children; ASD = autism spectrum disorder; WASI = Wechsler Abbreviated Scale of Intelligence; TB = time-based; CWI = Color Word Inference; SCQ = Social Communication Questionnaire; VF = Verbal Fluency; MWV = Monday to Wednesday version; TSV = Thursday to Saturday version; AUS = Australia; CG = control group; PM = Prospective Memory.

**Supplementary Table 3**

*Psychometric properties of the questionnaires included in the review.*

| PSYCHOMETRIC PROPERTIES | PRMQ-C (2014) | CFTQ (2020) |
| --- | --- | --- |
| Structural Validity | - | One factor: future-oriented cognition |
| Internal Consistency | Total Scale α = .93  PM Subscale α = .91 | Total Scale α = .93  PM subscale α = .83-.84 |
| Split-half Reliability | - | - |
| Inter-rater Reliability | - | - |
| Test-Retest | - | Total Scale r = .89  Subscales r = .74-.88 |
| Criterion Validity | CyberCruiser-II TBT (r =.318, p <.01)  Super Little Fisherman EBT (r =-.309, p <.01) | BRIEF-P (memory items) – PM Subscale r = -.32 (p = .002) |
| Convergent Validity | WASI (r = -.246, p <.05) Conner’s 2AI (r = .593-.743, p < .01) | - |
| Predictive Validity | - | - |
| Between-group Analysis | ADHD PM score > CG PM score (p < .05) | - |

*Note.* PRMQC = Prospective Retrospective Memory Questionnaire for Children; PM = Prospective Memory; WASI = Wechsler Abbreviated Scale of Intelligence; Conner’s 2AI = Conner’s 2 ADHD Index Rating Scales; TBT = time-bases task; EBT = event-based task; CFTQ = Children’s Future Thinking Questionnaire; BRIEF-P = Behavior Inventory of Executive Functions for Parents; ADHD = attention deficit hyperactivity disorder; CG = Control Group
